# Identification of *Mycobacterium avium* subsp. *hominissuis* secreted proteins using an in vitro system mimicking the phagosomal environment

**DOI:** 10.1186/s12866-016-0889-y

**Published:** 2016-11-09

**Authors:** Jessica J. Chinison, Lia Danelishvili, Rashmi Gupta, Sasha J. Rose, Lmar M. Babrak, Luiz E. Bermudez

**Affiliations:** 1 Department of Biomedical Sciences, College of Veterinary Medicine, Oregon State University, Corvallis, OR USA; 2 Department of Microbiology, College of Science, Oregon State University, Corvallis, OR USA

**Keywords:** Mycobacterium avium, Macrophages, Secretion, Proteins, Cytoplasm, Phagosome metal concentrations

## Abstract

**Background:**

*Mycobacterium avium* subsp. *hominissuis* is a common intracellular pathogen that infects patients with HIV/AIDS and cause lung infection in patients with underlying lung pathology. *M.avium* preferably infects macrophages and uses diverse mechanisms to alter phagosome maturation. Once in the macrophage, the pathogen can alter the host cellular defenses by secreting proteins into the cytosol of host cells, but despite considerable research, only a few secreted effector proteins have been identified. We hypothesized that the environmental cues inside the phagosome can trigger bacterial protein secretion. To identify *M. avium* secretome within the phagosome, we utilized a previously established in vitro system that mimics the metal ion concentrations and pH of the *M. avium* phagosome.

**Results:**

*M. avium* was exposed to phagosome metal concentrations for different time points and exported proteins were profiled and analyzed against bacterial proteins secreted in the culture medium. Mass spectrometric analysis of the secreted proteome identified several proteins, of which 46 were unique to bacteria incubated in the metal mixture. Ten of potential effectors were selected and secretion of these proteins was monitored within *M. avium* infected mononuclear phagocytic cells using the beta-lactamase FRET-based reporter system. In addition, pull-down assay was performed for secreted calmodulin-like protein MAV_1356 protein to evaluate for eukaryotic target. All examined *M. avium* proteins were secreted into the macrophage cytosol, and gene expression analysis suggested that the metal environment likely stimulates secretion of pre-made proteins. Further investigation of bacterial secreted MAV_1356 protein, lead to the observation that the MAV_1356 interacts with the host proteins Annexin A1 and Protein S100-A8.

**Conclusions:**

We established an in vitro system for the study if proteins secreted intracellularly, and revealed that the metal mixture mimicking the concentration of metals in the phagosome environment, triggers protein secretion.

## Background

*Mycobacterium avium* subspecies *hominissuis* (*M. avium*), an opportunistic pathogen found ubiquitously in the environment, is known to infect different mammalian hosts [1, 2]. Its transmission to humans occurs mainly through the gastrointestinal or respiratory routes [1]. *M. avium* is capable of infecting different cell types, but preferentially resides within macrophages [3]. Like *Mycobacterium tuberculosis*, *M. avium* survives and replicates within macrophages by blocking phagosome maturation and the fusion between the phagosome and lysosome [4], but many steps and mechanisms involved are still incompletely understood. The pathogen affects the transport of acid into the phagosome, as well as directly interferes with the trafficking of molecules associated with phagosome maturation in the cytosol [5].

Environmental cues, including metal ions present in the bacterial environment, can influence or control bacterial gene expression in general, including virulence factors. In *Salmonella enterica* spp. *enterica* serovar Typhimurium, the expression of the PhoP/PhoQ and the PmrA/PmrB, two component regulatory systems, are controlled by single elements in the environment [6]. The PhoP/PhoQ system responds to the magnesium concentration inside phagosomes, whereas the PmrA/PmrB system is regulated by iron level [6, 7]. Upon sensing environmental factors, such as the presence of antimicrobial peptides and different cations levels, both systems promote the expression of different genes such as *pagP*, *lpxO*, *pagL*, *pbgP* and *ugd*, resulting in several modifications on the bacterial lipopolysaccharide structure and the resistance to polymyxin [7, 8]. In *Yersinia*, the activation of the type III secretion system (T3SS) and the secretion of Yop effector proteins are dependent on the concentration of calcium. At a low calcium concentration, *Yersinia* T3SS delivers YopH and YopE outside of the bacterial cells, leading to disruption of the cytoskeleton and consequently prevention of phagocytosis [9].

The macrophage intraphagosomal metal composition of virulent and avirulent mycobacterial species has been described [10]. Using a hard X-ray microprobe, changes in iron, chlorine, calcium, potassium, manganese, copper and zinc concentrations inside the phagosome of macrophages infected either with *M. smegmatis*, *M. avium* or *M. tuberculosis* were documented. For example, diminished iron content was detected in the phagosomes of macrophages infected with *M. smegmatis* at different periods of time compared with macrophages infected with either *M. avium* or *M. tuberculosis* [10]. Following this finding, an in vitro system mimicking the single elements of the intracellular environment of the phagosome was developed [11]. A metal mixture was created, reproducing the concentrations of metal ion and pH of the phagosome at 1 and 24 h post-infection. Upon *M. avium* exposure to the 24 h metal mixture, expression was observed of a significant number of mycobacterial genes known to be upregulated within macrophages and in vivo. In addition, exposure to the combination of potassium chloride, calcium chloride and manganese chloride was sufficient to partially induce “the intracellular mycobacterial phenotype” associated with efficient uptake by surrounding macrophages once the bacteria escapes the macrophage [11]. These results demonstrate that this in vitro system could become a useful tool to study host-pathogen interaction.

Secretion of effector proteins leads to change in the pathogen environment and alterations of signal transduction and traffic mechanisms in the phagocytic cells. Many intracellular pathogens such as *M. tuberculosis*, Legionella, Coxiella, Salmonella and others, have been shown to depend on secreted molecules to control their environment inside the host for survival and replication [12–15]. There is substantial evidence that *M. avium* interferes with the macrophage function from inside of the vacuole [5, 16], and the expression of proteins on the bacterial surface or the export of effectors into the vacuole and/or the cytoplasm is manner to interact with the phagocyte.

Since protein secretion by the bacteria can certainly be a response to an environmental stimulus, we hypothesized that metals in the macrophage vacuole can have significant influence on secretion of pathogen-derived proteins. Here, we established an in vitro system for studying *M. avium* protein secretion and revealed that the metal mixture triggered protein secretion process.

## Methods

### *M. avium* and macrophage cultures

*Mycobacterium avium* subspecies *hominissuis* 104 (*M. avium* 104) was isolated from the blood of an AIDS patient and cultured on Middlebrook 7H10 agar supplemented with 10 % oleic acid, albumin, dextrose and catalase (OADC, Hardy Diagnostics, Santa Maria, CA) at 37 °C for 7 days. Human THP-1 cells were obtained from the American Type Culture Collection (Manassas, VA), cultivated in RPMI-1640 medium (Cellgro, Manassas, VA) supplemented with 10 % heat-inactivated fetal bovine serum, 2 mM L-glutamine and 25 mM HEPES, and incubated at 37 °C with 5 % CO_2_.

### *M. avium* exposure to metal mixture

The 24 h metal mixture used in this study was prepared as previously published [11] and is shown in Table 1. Freshly grown *M. avium* was resuspended in PBS, inoculated into Middlebrook 7H9 broth supplemented with OADC, and incubated at 37 °C for 5 days under 200 rpm of rotation. Unless noted specifically, all future mentions of 7H9 and 7H10 will be supplemented with 10 % OADC. After centrifugation at 2000 × *g* for 20 min, the bacterial pellet was washed, and then resuspended in 7H9 broth (without OADC). After passing the suspension 10 times through a 22-gauge needle to disperse clumped bacteria, the optical density (OD) was measured and adjusted to McFarland standard #1 (3 × 10^8^ CFU/ml). *M. avium* (3 × 10^7^CFU/ml) were inoculated into 7H9 broth or the 24 h metal mixture, and incubated shaking at 37 °C for either 4 or 24 h. The original inoculum was confirmed with serial dilution and plating for CFU. After 4 and 24 h post-exposure, bacterial viability was assessed with fluorescent microscopy using a commercially available live/dead BacLight kit (Molecular Probes, Eugene, OR). The pH was measured at both time points and shown to be 5.8. After 4 or 24 h incubation, the cultures were centrifuged at 2000 × *g* for 30 min at 4 °C to collect supernatants.

**Table 1:** The elemental mixtures reproducing metal concentrations and pH of 1h and 24h *M. avium* phagosome

Supplement	Elemental mix added to 500 ml of Middlebrook 7H9 broth	
	1h (pH 6.6)	24h (nM, pH 5.8)
1 M potassium chloride	14.7 ml	0.925 ml (1.79)
1 M calcium chloride	2 ml	1.25 ml (2.42)
1 M manganese chloride	5.9 ml	11.9 ml (23)
1 M copper sulfate	1.85 μl	5.5 μl (0.01)
1 M zinc chloride	33 μl	58.7 μl (0.11)
0.25 M ferric pyrophosphate	288 μl	2 ml (0.97)
1 M nickel chloride	5 μl	5 μl (0.01)

### Secreted proteome and mass spectrometry

Metal or 7H9 exposed supernatants were filtered through a 0.2 μm syringe filter and size fractionated using 300 and 3kDa size-exclusion centrifugal filters (Pall, Ann Harbor, MI). The concentrated protein fraction between 3 and 300kDa was resolved with SDS-PAGE through a 12 % gel following standard protocols until the samples travelled 1cm through the gel. The gel was stained with Coomassie brilliant blue and then excised. Samples were distained and processed for the Protease Max in-gel trypsin digestion (Promega, Madison WI), following the manufacturer protocol. Digested proteins were further purified with a Michrom Peptide CapTrap column and C18 column (Agilent Zorbax 300SB-C18, 250 × 0.3mm, 5μm). Samples were sequenced and analyzed in the Mass Spectrometry Facility at Oregon State University using the LC-MS/MS with a Thermo LTQ-TF MS coupled to a Waters nanoAcquity UPLC system. Raw data was blasted against the Proteome Discoverer v1.3.0. and Mascot v2.3 database and Scaffold was used for search data compilation and data evaluation. The assay was repeated independently two times, and we only considered the proteins identified in both assays (96 % of the total proteins).

### Cloning of *M. avium* genes into beta-lactamase reporter vector

*M. avium* 104 genomic DNA was extracted as previously described [17, 18]. Primers specific to 10 *M. avium* 104 genes were designed for PCR amplification and are listed in Table 2. Amplicons were processed for electrophoresis on 1 % agarose gel, and purified with the QIAquick PCR purification kit following the manufacturer’s instructions (Qiagen, Venlo, Netherlands). The genes of interest were cloned into BamHI and EcoRI sites of the pMV261-BlaC vector containing the beta-lactamase gene lacking the signal sequence. Resulted constructs were propagated in *E.coli* grown in LB broth supplemented with kanamycin 50 μg/ml.

**Table 2:** Primers used in the study

Gene Name	Experiment	Primer	Sequence (5′ to 3′)
MAV_0502	BlaC	F	GGATCCATGCTCGACGCCGTG
		R	GAATTCTTGCCCCGCAATGAGAACG
	RT-PCR	F	GTGAGCTGCAATGTCGGATG
		R	GCAGTACTGGTGCAGATCGT
MAV_0516	BlaC	F	GGATCCATGCCACAGGGAACTGTG
		R	GAATTCCAGGGAGCGGACTCCG
	RT-PCR	F	ACAGGGAACTGTGAAGTGGTT
		R	GATTTCGAACTCGACCTTCTGG
MAV_0628	BlaC	F	GGATCCATGAGCACATCCAACACAGTC
		R	GAATTCGGGCGGGCAGGAGTG
	RT-PCR	F	TCGCCGAGGGGTTACTCA
		R	AGGTAGTCGATGACGTTGCC
MAV_1356	BlaC	F	GGATCCATGGCGGCGATGAAG
		R	GAATTCGCTGGAGCTCGTGACG
	RT-PCR	F	ATGGCGGCGATGAAGCC
		R	CTTGAGTTCGTCACGGAGGG
MAV_4394	BlaC	F	GGATCCGTGAAAACTCACCGGATCG
		R	GAATTCGACGAGAGGTGCTTGCGAAG
	RT-PCR	F	CCTACGGGGTCAACTATGCC
		R	CACGATGTCCATCACCGAGG
MAV_1419	BlaC	F	GGATCCAATGTTCTCGCGCCGCATCATCACC
		R	GAATTCGGCGGGCTGGCCGGAGAATTGCGGG
	RT-PCR	F	CGATGAGATGTTCCTCGCCC
		R	
MAV_0398	BlaC	F	GGATCCAATGACCATGCGGATCACGCGGGTTTGCCGGGCGGT
		R	GAATTCTTGACCCAGCGCGTTTTGCATGGCCCCGG
	RT-PCR	F	GACGAGAACTGGACCAAGCA
		R	TATTGACCCAGCGCGTTTTG
MAV_1177	BlaC	F	GGATCCATGAGCATCAACTACCAGTTC
		R	GAATTCGGCCCAGCTGGACC
	RT-PCR	F	ACTACCAGTTCGGCGATGTC
		R	CGACCCAACTGGGTGATGAA
MAV_1178	BlaC	F	GGATCCATGGCAACACGTTTTATGACTG
		R	GAATTCGCTGCTGAGGATCTGCTG
	RT-PCR	F	TTATGACTGACCCGCACGAA
		R	GTTGACGATGTTGCGGAAGG
MAV_4077	BlaC	F	GGATCCAATGGGGGACCAGCAGAGCGGGCCGCAGGAA
		R	GAATTCTTGGTTCTTCTTCTGGCGCT
	RT-PCR	F	GAAGGGCAAGGCCAAGGA
		R	TCTTCTTCTGGCGCTCCTC

### *M. avium* transformation and colony screening

*M. avium* competent cells were prepared as followed: 3 centrifugal washes at 2000 × *g* for 15 min with a chilled wash buffer constituted of 10 % glycerol and 0.1 % Tween-20. The final pellet was resuspended into 10 % glycerol. Cells were electroporated with plasmid DNA and recovered for 4 h at 37 °C in 7H9 broth before being plated on 7H10 agar containing 400 μg/ml of kanamycin. After 10 days incubation at 37 °C, colonies were screened with PCR for the presence of kanamycin using the following primers: Full_Kan_For 5′ ATATTCAACGGGAAACGTCTTG 3′ and Full_Kan_Rev 5′CATCGAGCATCAAATGAAACTG 3′. The PCR program was as follows: 95 °C for 5 min, 35 cycles of 95 °C for 30 s, 60 °C for 30 s, 68 °C for 1 min and a final 68 °C for 5 min. Positive colonies were grown in 7H9 broth and 400 μg/ml of kanamycin. After 5 days incubation at 37 °C, the bacterial cultures were used for uptake, survival and beta-lactamase assays. Prior to the following experiments, the bacterial cultures were briefly vortexed, passed 10x through a 22-gauge needle to disperse clumps, and allowed to sediment for 5 min. Only the top half of the suspension was used as the inoculum.

### Beta-lactamase reporter system

THP-1 cells were treated with 10 ng/ml of phorbol-12-myristate-13-acetate to differentiate monocytic cells into macrophages, seeded at 80 % confluency into the 96-well plate (Greiner Bio-One, Monroe, North Carolina) and incubated for 24 h. Next day, culture media was replenished and THP-1 cells were rested additional 48h. Differentiated macrophages were infected with *M. avium* constructs and controls for 2 h at a multiplicity of infection 10 bacteria to 1 cell. The extracellular *M. avium* was removed by washing cells three times with HBSS. After carrying out intracellular infection for 3 days, infected cells were reloaded with CCF2-AM (Invitrogen), a beta-lactamase substrate, at 20 μg/ml in RPMI medium. Fluorescent measurements were taken every 15 min for 2 h using an Infinity 200 fluorometer (Tecan, Männedorf, Switzerland) with two filter sets: Excitation 405 ± 10 nm/emission 460 ± 20 nm and excitation 405 ± 10 nm/emission 530 ± 15 nm.

### Fluorescent microscopy

THP-1 cells were also examined using an inverted fluorescent microscope (Leica, Wetzlar, Germany) with the proper CCF2-AM filter sets listed above. Images were captured using a Retiga-2000R CCD camera and QCapture Pro software (QImaging, Surrey, BC, Canada).

### Uptake and survival assay

THP-1 monocytes were differentiated as described above and seeded in 96-well plates at 80 % confluence. Macrophages were infected with ten *M. avium* constructs and controls at an MOI of 10. After 2 h of infection, cells were washed three times with HBSS, lysed, serially diluted and plated on 7H10 agar to determine bacterial colony forming unites (CFUs) and to calculate the *M. avium* invasion rate. To assess survival, cells were lysed and plated at 3 days post-infection. The infection/survival rates were compared to control groups.

### RNA extraction and purification

*M. avium* experimental and control constructs were exposed to 1- or 24-h metal mixture and 7H9 broth for 1 and 24 h as a control. A two-step differential centrifugation was performed to separate the bacteria from the metals. After the first centrifugation at 55 × *g* for 15 min at 4 °C, the supernatant (containing the bacteria) was decanted and then centrifuged at 2000 × *g* for 20 min at 4 °C to collect bacteria. RNA was extracted from the resulting bacterial pellets as previously described [19]. Briefly, the pellet was resuspended into a buffer containing 500 mM of sodium acetate (pH = 4), 1 % triton X-100 and 0.6 % sodium dodecyl sulfate. The suspension was mixed with 0.1mm glass beads and homogenized at 4800 oscillations/minute in a bead-beater 6 times for 20 s and kept on ice in between each step. Samples were centrifuged for 5 min at 10,000 × *g* and then the supernatant was subjected to two separate phenol:chloroform extraction series with an ethanol precipitation and DNase treatment between the two extraction series. RNA was ethanol precipitated after the second extraction series, and resuspended into RNase-free H_2_O. This final resuspension was DNase treated a second time with the Turbo DNase system (Life Technologies) that efficiently removes the enzyme after digestion. The concentration and purity of bacterial RNA was measured using a NanoDrop 2000 instrument (Thermo Scientific, Wilmington, DE). PCR was used to check for residual DNA by amplification of the 23s RNA gene and was negative for all RNA samples.

### Reverse-transcription PCR analysis

cDNA was produced by reverse-transcription of the RNA using iScript reverse transcriptase (Bio-Rad, Hercules, CA), following the manufacturer protocol. cDNA was amplified using the primers listed in Table 2. The PCR program was as follows: 95 °C for 5 min, 31 cycles of 95 °C for 30 s, 59 °C for 30 s, 68 °C for 30 s and a final 68 °C for 5 min. The following primers, 23S_F 5′ GTAGCGAAATTCCTTGTCGG 3′ and 23_R 5′ TTCCCGCTTAGATGCTTTCAG 3′, recognizing the housekeeping gene, 23S RNA and the following PCR settings, 95 °C for 5 min., 95 °C for 30 s., 59 °C for 30 s., 68 °C for 1 min., for 31 cycles, were used to amplify the 23S RNA gene, which acted as a control in this experiment. cDNA amplicons were visualized with 1 % gel electrophoresis.

### Protein-protein interaction

To create the MAV_1356 overexpression construct, the gene was amplified form the *M. avium* 104 chromosomal DNA using FideliTaq PCR master mix (Affymetrix, Santa Clara, CA) and the PCR-generated fragment was cloned into the pET6xHN-N vector (Clontech). The protein was expressed in *E. coli* strain BL21 (DE3) and purified according to the manufacturer’s instruction (Clontech). Alternatively, THP-1 cells were harvested from the 75mc^2^ flask, washed and mechanical lysed in PBS. Cell lysate was cleared by centrifugation at 500 rpm for 10 min, filtered through 0.2 μm filter and labeled with the sulfo-NHS- LC biotin (Thermo Scientific) as previously described [20]. The purified MAV_1356 protein and the total protein extract from THP-1 cells were loaded to the column of His60 nickel resin and incubated for overnight at 4 °C with rotation. The columns that contained just THP-1 protein extract with no bacterial protein exposure served as a control for non-specific binding to nickel resin. Following day, columns were washed according to the manufacturer’s protocol (Clontech) to remove unbound host cell proteins and MAV_1356 with possible bound host proteins were eluted with elution buffer. The samples were boiled in Laemmli sample buffer and separated onto SDS-PAGE gel, transferred to nitrocellulose membrane and blocked with 3 % Bovine Serum Albumin (BSA). Membrane was probed with streptavidin antibody (Li-Cor Biosciences, Inc) at a dilution of 1:1000 for 1 h and visualized with the Odyssey Imager (Li-Cor). The proteins of interest were exercised from the gel and sent for the mass spectrometric analysis at the Oregon State University Proteomics facility.

### Statistical analysis

A Student’s *t* test used to determine significance between experimental groups and control groups. A *p*-value less than 0.05 was considered significant. GraphPad Prism version 6.0 was used for statistical analysis and graph creation.

## Results

### *M. avium* secretes unique proteins when incubated in a metal mixture mimicking the environment of the phagosome

Studying protein secretion inside host cells can be technically challenging and complex. For these reasons, an in vitro system mimicking metal concentrations in the vacuole environment of the macrophage and a proteomic-based approach were used to identify secreted proteins of *M. avium* strain 104. A total of 55 secreted proteins were identified when the bacteria were exposed for 4h and 24h to metal concentrations found in 24h *M. avium* vacuole and in the 7H9 culture supernatants of *M. avium* (Table 3). From 55 identified secreted proteins, 46 were uniquely associated with the 24 h metal mixture and absent in the 7H9 broth control confirming that bacteria in broth does not secrete many proteins during the short period of observation. Twenty proteins out of 46 were more abundant during 24 h of exposure than 4 h of incubation. Eighteen additional proteins were found only at 24 h of exposure. Only 2 proteins were found more abundant at 4 h than 24 h and 2 were unique to the 4-h exposure. Four proteins were found at equal levels regardless of metal exposure period. Majority of known secreted proteins including MAV_2816 and MAV_0214, which represented antigen 85-B and antigen 85-A respectively, were identified in metal-exposed condition at both time points. One third of the identified secreted proteins were putative uncharacterized proteins. Ten hypothetical genes, encoding for secreted proteins unique to the metal exposure, were chosen for further study. Criteria used to select this 10 candidates included proteins that had interesting motifs, were small in size (characteristic of many secreted proteins) and homologous to *M. tuberculosis* proteins shown to be secreted, and some unknown not yet identified as to be secreted in macrophages (Table 4).

**Table 3:** Proteins identified from *M. avium* 104 supernatant during exposure to metal mixture of the concentrations encountered in phagosome or control 7H9 broth

		Peptide Abundance			
*M. avium* protein	Identity	4h 7H9	4h metals	24h 7H9	24h metals
MAV_2017	Low molecular weight antigen	0	3	0	7
MAV_1178	Hypothetical protein	0	3	0	6
MAV_2193	Acyl carrier protein	1	4	5	6
MAV_4366	10 kDa chaperonin	1	2	5	3
MAV_1177	Hypothetical protein	0	2	0	3
MAV_4283	Probable Cutinase Cut3	0	2	0	8
MAV_0628	Hypothetical protein	0	2	0	3
MAV_3743	Elongation factor Ts	0	2	1	3
MAV_2859	ModD protein	0	1	0	4
MAV_0406	Glycerol kinase	0	2	0	5
MAV_4394	Serine esterase, Cutinase family protein	0	1	0	4
MAV_4507	50S ribosomal protein	0	1	0	3
MAV_2816	Antigen 85-B	0	3	0	3
MAV_2880	Malate synthase	0	3	0	3
MAV_1198	Acetyl-CoA acetyltransferase	0	2	0	4
MAV_5271	Fructose-bisphosphate aldolase class-I	0	2	0	3
MAV_4079	Hypothetical protein	0	2	0	2
MAV_4583	Hypothetical protein	0	1	0	3
MAV_4695	Hypothetical protein	0	1	1	2
MAV_0182	Superoxide dismutase	0	2	0	1
MAV_0214	Antigen 85-A	0	1	0	2
MAV_0502	Hypothetical protein	0	1	0	3
MAV_0516	Conserved domain protein	0	1	0	3
MAV_4240	Two-component response regulator	0	1	0	3
MAV_4130	Immunogenic protein MPT64	0	0	0	3
MAV_3875	Electron transfer flavoprotein	0	0	0	3
MAV_1096	Protease	0	1	0	2
MAV_4274	PPE family protein	0	1	2	1
MAV_4077	Hypothetical protein	0	2	0	2
MAV_1356	Hypothetical protein	0	0	1	2
MAV_3557	Hypothetical protein	0	1	0	3
MAV_0239	Hypothetical protein	0	1	0	2
MAV_2019	Low molecular weight antigen MTB12	0	0	0	4
MAV_2763	Hypothetical protein	0	1	0	2
MAV_1419	Hypothetical protein	0	2	0	1
MAV_4707	60 kDa chaperonin 2	0	0	2	1
MAV_1204	Transcription elongation factor	0	0	0	2
MAV_0589	Hypothetical protein	0	0	0	3
MAV_2725	Peptidyl-prolyl cis-trans isomerase	0	0	0	3
MAV_3539	Hypothetical protein	0	0	0	3
MAV_4986	ErfK/YbiS/YnhG family protein	0	0	0	3
MAV_4489	Elongation factor Tu	0	0	3	0
MAV_0435	Anti-sigma factor antagonist	0	0	0	2
MAV_2795	Hypothetical protein	0	0	0	2
MAV_0398	Hypothetical protein	0	0	0	2
MAV_0426	Intracellular protease	0	0	0	2
MAV_2345	Wag31 protein	0	0	0	2
MAV_2770	Thiol peroxidase	0	2	0	0
MAV_2905	PPE family protein	0	0	2	0
MAV_3158	Response regulator	0	0	0	2
MAV_3640	Antibiotic biosynthesis monooxygenase	0	0	0	2
MAV_3876	Electron transfer protein, beta subunit	0	0	0	2
MAV_4339	Phospoglycerate mutase family protein	0	0	0	2
MAV_4936	Succinate-semialdehyde dehydrogenase	0	0	0	2
MAV_1380	Malate dehydrogenase	0	2	0	0

**Table 4:** *M. avium* genes selected for intracellular confirmation

Gene	Function	Motif	M.tb homology	Secreted in M.tb
MAV_1178	Hypothetical protein	WXG100: ESAT-6 Type VII secretion target	Rv2347c	Unknown
MAV_1177	Hypothetical protein	WXG100: ESAT-6 Type VII secretion target	Rv2346c	Y
MAV_0628	Hypothetical protein	MHB: coiled-coil molecule that binds free haem in mycobacterial cytoplasm to deliver it to membrane proteins for shuttling through the membrane	None	N
MAV_4394	Hypothetical protein	Serine esterase, Cutinase family protein	Rv3451	Unknown
MAV_0502	Hypothetical protein	None	None	N
MAV_0516	Cold shock protein	CSPA: probably involved in cold acclimation process	Rv3648c	Y
MAV_4077	Hypothetical protein	CsbD: bacterial general stress response protein	None	N
MAV_1356	Hypothetical protein	98 % Homologues to *M. tuberculosis* calmodulin*-*like protein. DUF: proteins with unknown function restricted to Actinobacteria	Rv1211	Y
MAV_1419	Hypothetical protein	DUF: proteins with unknown function restricted to Actinobacteria	None	N
MAV_0398	Hypothetical protein	PkhH_C: found as the periplasmic domain of the bacterial protein kinase PknH	Rv3705c	Y

### Proteins secreted in the metal mixture are also exported during intracellular infection

**Fig. 1:**
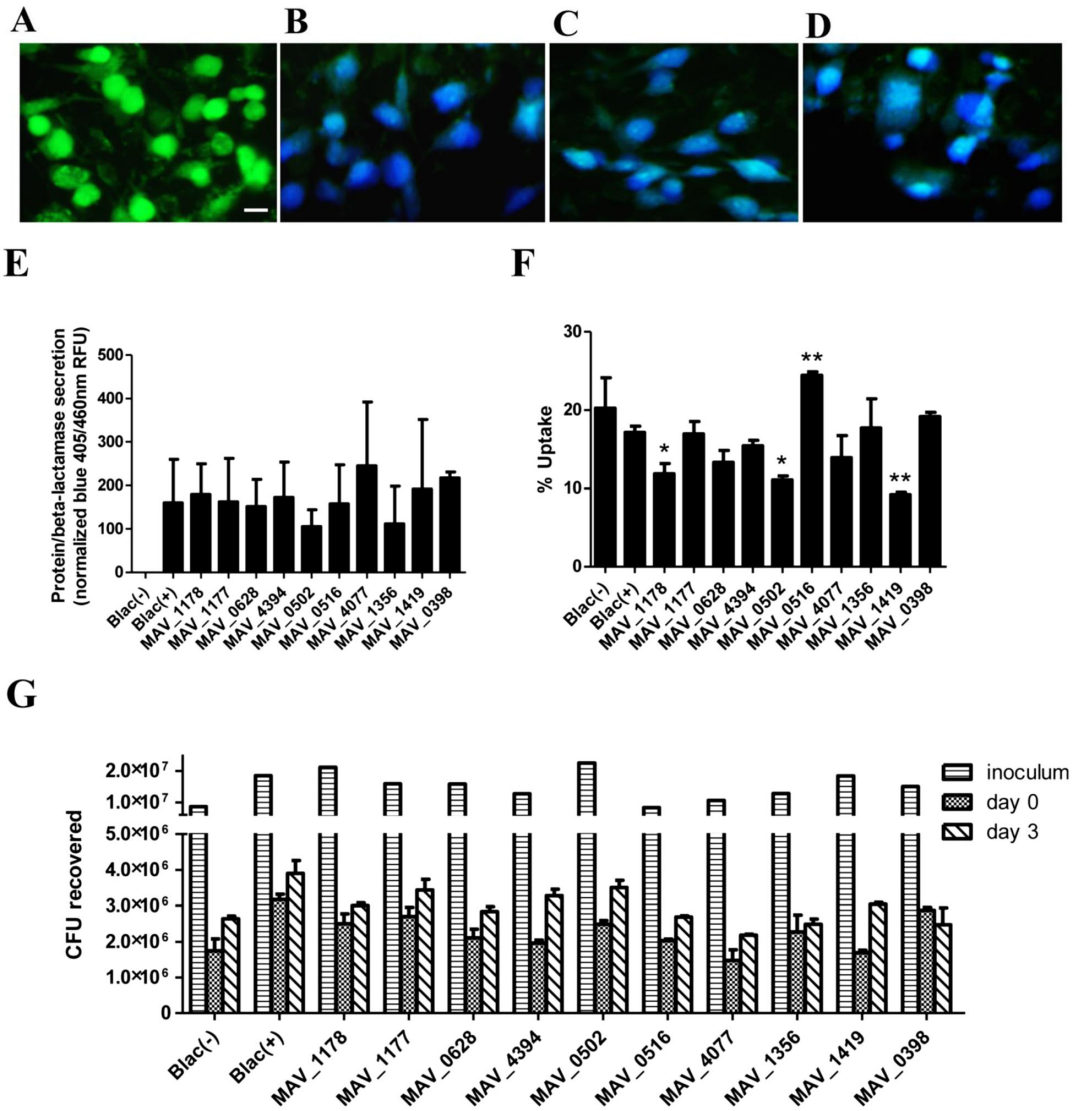
*M. avium* secreted proteins within THP-1 macrophages. Differentiated THP-1 cells were infected with *M. avium* gene constructs fused to a beta-lactamase without secretion signal. After 3 days of infection, FRET-based substrate, CCF2-AM, was loaded into the cells and a shift in fluorescence was observed microscopically (**a**–**d**) and quantitatively (**e**). **a** *M. avium* transformed with pMV261-BlaC(-) negative control. **b** *M. avium* transformed with pMV261-BlaC(+) positive control. Since all 10 of the tested secreted protein constructs produced a color change, we randomly picked MAV_4077 (**c**) and MAV_0516 (**d**). Bar, 10μm. **e** Calculated and normalized fluorescence ratios of the 10 tested protein constructs from the same wells where the fluorescent microscopy was conducted. **f.** Infection rate was calculated by bacterial CFU at 2 h of initial infection divided by the infection inoculum and normalized for small differences in inoculum between the constructs. **g** CFU of *M. avium* recovered during 3 days of infection. Bars represent mean values ± standard deviation. Data shown is representative of three biological replicates. Statistical comparisons: *P <0.05 and **P <0.01 when compared to pMV261-BlaC(+)

To investigate if the secretion of selected proteins identified from the metal mixture proteome occurred during intracellular infection as well, a beta-lactamase reporter system was utilized. Constructs were made by fusing selected *M. avium* genes to a beta-lactamase without a secretion signal, and transformed into *M. avium* strain 104, which doesn’t encode a functional native beta-lactamase. THP-1 cells were infected with the different *M. avium* constructs, and after 3 days a mammalian FRET-based substrate was loaded into the cells. The substrate cleavage in the cytosol of macrophages would happen only if the *M. avium*/beta-lactamase fusion protein is secreted and present in the cytoplasm producing the differential fluorescent signal inside the host cells [17, 18]. In THP-1 cells infected with the *M. avium* pMV261-BlaC(-) negative control (beta-lactamase construct without signal peptide) CCF2 substrate cleavage does not occur and they fluorescent green (Fig. 1a); whereas in THP-1 macrophages infected with the *M. avium* pMV261-BlaC(+) positive control (beta-lactamase with signal sequence) substrate cleavage results in robust fluorescent changes from green to blue (Fig. 1b). Using this reporter system, intracellular secretion of ten selected proteins was confirmed. Since the fluorescent microscopy pictures looked similar for all 10 constructs, MAV_4077-BlaC and MAV_0516-BlaC were randomly selected and are shown in Fig. 1c–d, respectively. In addition, the secretion of MAV_1177, known protein to be secreted by *M. tuberculosis*, served as a positive control.

The amount of blue fluorescence in the cells was quantified using a fluorometer and directly correlates with the amount of secreted protein/beta-lactamase molecules. Majority of *M. avium* protein constructs elicited similar levels of blue fluorescence as the positive control. While MAV_0516 and MAV_1356 produced less protein secretion, MAV_4077 correlated with more protein export (Fig. 1e). To control for differences between the infection kinetics, bacterial uptake and survival at 3 days post-infection were determined by recording the CFU counts (Fig. 1f and g, respectively). The uptake of MAV_1178-BlaC, MAV_0502-BlaC and MAV_1419-BlaC were significantly impaired compared to the control pMV261-BlaC(+). In contrast, the uptake of MAV_0516 was significantly increased over the pMV261-BlaC(+) control. Regardless of the difference, 9 of the 10 *M. avium* constructs were able to survive and grow at 3 day post-infection with the exception of MAV_0398-BlaC that showed a decrease in survival (Fig. 1g).

### Selected *M. avium* proteins are pre-synthesized upon exposure to the metal mixture

**Fig. 2:**
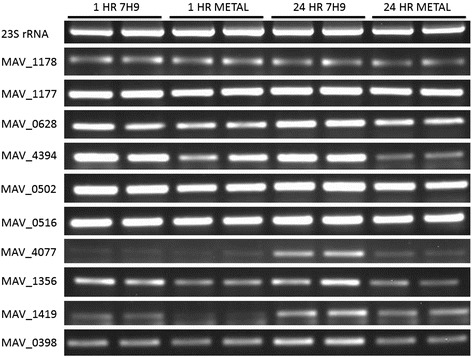
*M. avium* gene expression levels during metal exposure. *M. avium* 104 was incubated in either 7H9 broth or the 24 h metal mixture for 4 and 24 h. The total RNAs for the10 confirmed secreted proteins were extracted and analyzed for gene expression by RT-PCR

To determine whether *M. avium* exposure to the metal mixture would trigger gene expression, we performed a reverse-transcription PCR (RT-PCR) analysis for the 10 confirmed secreted proteins [19]. *M. avium* 104 was exposed to 24-h metal mixture and control 7H9 broth for either 1 or 24 h and gene expression levels were quantified by the RT-PCR. As shown in the Fig. 2, similar gene expression levels were observed between the metal and 7H9 broth exposed bacteria at both time points. Since *M. avium* exposure to the metal mixture for 1 or 24 h did not changed the gene expression levels for any of the 10 genes selected, it suggests that these proteins were already pre-synthesized and the exposure to metals triggered/activated the protein secretion mechanism.

### The *M. avium* calmodulin-like protein interaction with host proteins

**Fig. 3:**
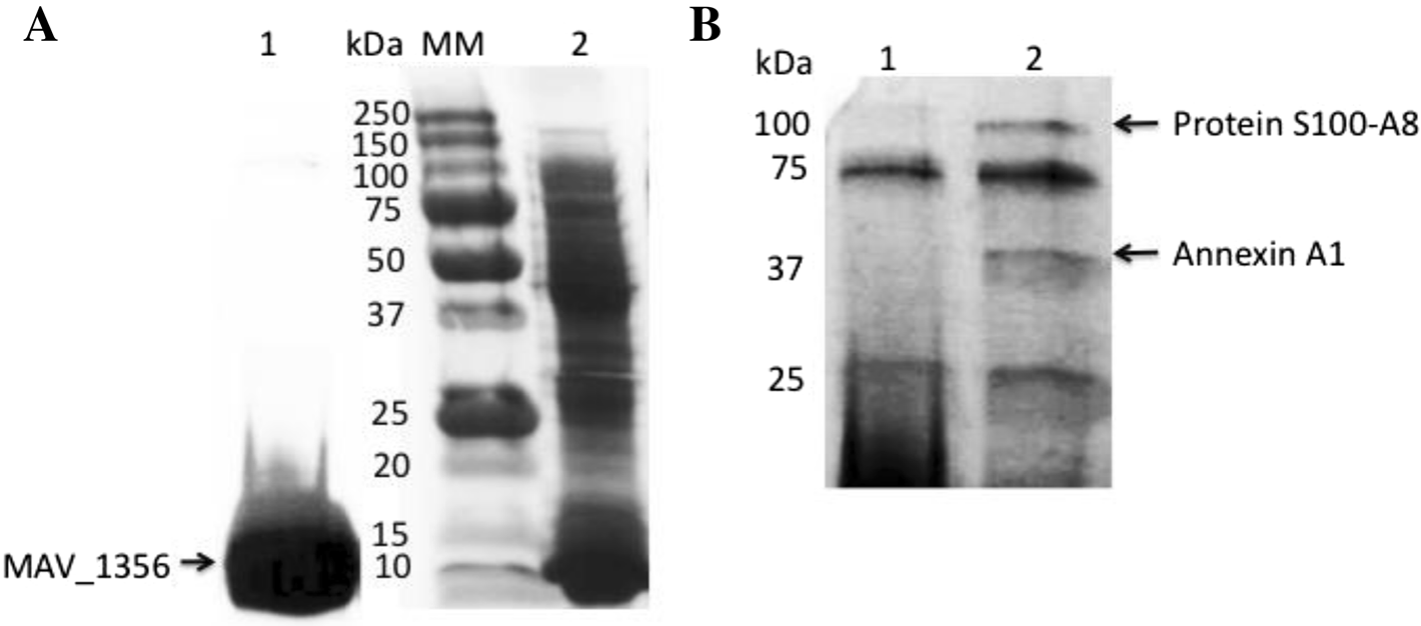
Host proteins interacting with MAV_1356. **a** Lane 1, The western blotting of the 6HN-MAV_1356 protein; Lane 2, The coomassie staining of the *E. coli* total protein extract overexpressing the MAV_1356 protein; MM, Molecular Marker. **b** Lane 1, the biotin-labeled host proteins nonspecifically binding to the His60 nickel resin (control group without bacterial protein); Lane 2, the biotin-labeled total protein extract of THP-1 cells were exposed to the recombinant MAV_1356 (experimental group) and the bound proteins were visualized with streptavidin antibody as described in the materials and methods; Both groups were subjected to the same procedures. The unique proteins identified just in experimental lane was exercised and sequenced. The MAV_1356 bound host proteins were identified as Annexin A1 and Protein S100-A8

The MAV_1356 protein was produced in *E. coli* using the pET Expression system and purified with the His-Bind resin chromatography (Clontech) as per the manufacturer’s instructions. The overexpression of MAV_1356 protein was confirmed in *E. coli* cell lysate by coomassie staining, and purified protein were visualized by western blotting using the 6XHN rabbit antibody (Clontech) (Fig. 3a). The mass spectrometric analysis was performed using the Thermo Fisher LTQ VELOS mass spectrometer. The unique bands bound to the bacterial MAV_1356 protein and present in the pull-down assay were identified as 38kDa Annexin A1 (P04083) and Protein S100-A8 (P05109) (Fig. 3b).

## Discussion

*M. avium* survives and replicates in macrophages and, as many of intracellular pathogens, requires to secrete an array of effector proteins to establish its intracellular niche and prevent the killing mechanisms of phagocytic cells. Phagosomes of *M.avium* in macrophages are initially large, but segment and split in one bacterium, one phagosome after hours following phagocytosis [4]. Virulent mycobacteria inhibit phagosome acidification [1, 4, 5]. In this study, we utilized an in vitro system that mimics the concentrations of single metals in the phagosome of *M. avium* infected macrophages [10]. Previous work demonstrated that the presence of specific metals influence bacterial gene regulation in vitro*,* and it is similar to the one observed with the intracellular *M. avium* residing in macrophages [11].

To investigate whether metals have a role in the signaling mechanism and can stimulate the secretion of *M. avium* proteins, we exposed bacteria to metal mixture with concentrations that are encountered inside of macrophage vacuole. Forty-six unique proteins identified by mass spectrometry were exported in the vacuole model, while none of these proteins were secreted in the culture medium without the metals. Out of the 46 proteins, some are known effectors to be secreted inside macrophages, while others represent novel observation. For instance, MAV_1177 and MAV_1178 are hypothetical proteins containing the WXG motif. These two proteins are ESAT-like proteins and have significant homology to Rv2346c and Rv2347c of *M. tuberculosis*, respectively. In *M. tuberculosis*, Rv2346c (esx0) and Rv2347c (esxP) are associated with the ESAT-6-CFP-10 complex [21, 22]. By using a beta-lactamase FRET system we were able to confirm that 10 out of 10 tested proteins were in fact secreted and transported to the cytosol of the macrophage. MAV_4077, MAV_0502, MAV_4394, MAV_1419 and MAV_0628 are hypothetical proteins with no homology to *M. tuberculosis* proteins. MAV_4394 is a protein with signal sequence and a cutinase motif. Seven cutinase-like proteins have been identified in *M. tuberculosis*, yet their functional diversity and immunological properties vary significantly [23, 24]. Two cutinase-like proteins, Rv1984c and Rv3452, have been shown to be secreted by *M. tuberculosis* [25]. Their function was qualified as lipases and esterases.

Among identified secreted proteins, MAV_1356 hypothetical protein, with homology to the Rv1211 calmodulin-like protein in *M. tuberculosis*, has attracted our attention. It has been demonstrated in *M. tuberculosis* that this protein blocks the phagosome-lysosome fusion by inhibiting the entry of the cytosolic Ca^++^ inside the vacuole and disrupting the downstream signals in macrophage mediated by Ca^++^ Calmodulin [26, 27]. Our findings suggest that *M. avium* MAV_1356 interacts with cytoskeleton protein Annexin A1, a functional linker between actin filaments and phagosomes [28], and supports possibility that this protein might have similar function as the *M. tuberculosis* Rv1211. Annexins are well known to be present on phagosomes. Annexin A1 is a factor that binds to phagosomes in presence of calcium and facilitate the interaction of F-actin in the phagosome membrane. Downregulation of Annexin A1 gene expression results in impaired phagosome fusion with intracytoplasmatic structures [28]. MAV_1356 was shown to bind to S10A8_HUMAN (calprotectin), a phagocyte protein that is a calcium-binding protein and plays a number of roles in the intracellular environment, such as modulation of tubulin-dependent cytoskeleton.

The fact is that the slow replicating bacteria require an extended periods of time to synthesize *de novo* proteins (in *M. avium* it takes approximately 30 min, Bermudez, personal communication). One of the major questions in pathogenesis of slow replicating bacteria including *M. avium* is that during the first encounter with the host, in order for the pathogen to establish the successful infection, it would need to have some sets of proteins already pre-synthesized and ready to interact with the host cells. Our observation that metals do not seem to regulate gene expression per se, but stimulate the secretion of pre-synthesized proteins, supports ths idea. It also raises the question that at least some of the bacterial secretion mechanisms may be under the control of single elements. The implication would be that if one interferes with metal transport into the phagosome, it might impact secretion of effectors with consequent modulation in virulence. It is interesting that the majority of the identified secreted proteins belong to the ESX-3 region of *M. avium* chromosome. In *M.* tuberculosis the region is associated with the control of iron and zinc homeostasis [29].

There is always the concern that proteins identified in the supernatant were generated by bacteria lysis. While it is certainly true, in our phagosome model, *M. avium* is exposed just for short time periods (4h or 24h) and our experimental setting completely differs from studies where mycobacterial secreted proteins are identified in the filtrate of bacterial culture grown for several weeks [30, 31]. In addition, the conformational study verified that all the 10 proteins that we selected from the metal mix are in fact truly secreted in *M. avium* infected macrophages. Limited number (just 55) of proteins found by proteomic analysis also suggests that no bacterial lysis has occurred.

In summary, we described an in vitro system mimicking the phagosome environment and identified some sets of *M. avium* secreted proteins. This provides a new approach that is applicable to other intracellular organisms. The observation of several hypothetical proteins being secreted in the macrophage cytosol will require additional studies to identify the possible macrophage targets and function.

## Conclusions

This study shows that an in vitro model representing the phagosome environment of the macrophage can be used to identify bacterial secreted protein relevant to infection. Although not all secreted proteins are identified, the model is quite efficient in unveil new secreted effectors.
